# T231. PALIPERIDONE LONG-ACTING INJECTABLE (LAI) IS ASSOCIATED WITH A LOWER INTAKE OF BENZODIAZEPINES AND A LOWER NUMBER OF ADMISSIONS COMPARED WITH OTHER LAIS IN A COHORT OF PATIENTS WITH SCHIZOPHRENIA

**DOI:** 10.1093/schbul/sby016.507

**Published:** 2018-04-01

**Authors:** Juan-Antonio Garcia-Carmona

**Affiliations:** Reina Sofia University Hospital

## Abstract

**Background:**

Schizophrenia is characterized by a chronic course that in most of cases requires continued and long-term treatment by the use of long acting injectables (LAI). Several LAI formulations have been available and increased the number of pharmacotherapeutic options for schizophrenia. However, there are no formal recommendations regarding the use of specific LAIs for the treatment because there are only a few available studies and even fewer studies assessing which LAI formulation is more effective or suppose an advantage. Many studies has been published regarding the prescription patterns of oral anti-psychotic and the transition to a LAI treatment in real-life settings but the patterns and predictors of use of different LAI formulations, and the effect of introducing new LAI drugs has drawn even less attention. The aim of this study was to analyze the anti-psychotic LAI prescription patterns during a 3-years follow-up period in Murcia, Spain, and to identify predictors of medication changes associated to the use of LAIs.

**Methods:**

we designed a non-interventional, naturalistic and retrospective study using all patients from the health department corresponding to the Reina Sofía University Hospital, Murcia, Spain who were diagnosed of schizophrenia and treated with long-acting injectables (LAIs) between the years 2015–2017. Data, pertaining to patients older than 18 years old, were extracted from electronic medical records. Demographic variables, the use of LAIs, the rates for antipsychotic polypharmacy, combined use of different antipsychotic classes with a special focus on atypical antipsychotics, and psychotropic polypharmacy using benzodiazepines, mood stabilizers, and other relevant drugs were identified, as well as, the number of admissions and suicidal attempts.

**Results:**

No statistical differences were observed regarding demographic variables between LAIs users. However, the number of admissions to a hospital or acute relapses where significantly lower (p<0.05) in the group treated with paliperidone LAI versus the others LAIs. In addition, our results showed that paliperidone, aripiprazole, zuclopentixol and riperidone LAIs are associated with 15.85; 47.86; 25 and 49.25 mg/day of diazepam, respectively. One way ANOVA showed that paliperidone LAI was associated with a significant (p<0.01) less intake of benzodiazepines when compared to others LAIs. ANOVA failed to show differences when the dose of oral anti-psychotics co-administered with LAIS were compared.

**Discussion:**

Polypharmacy is the most common pattern of antipsychotic use in this region of Spain. Use of atypical antipsychotics is extensive. Most patients receive psychiatric co-medications such as anxiolytics. Polypharmacy is associated with the use of aripiprazole or zuclopentixol and these groups of patients showed more admissions to a hospital. In contrast, paliperidone LAI group showed less admissions and less use of benzodiazepines. Our results indicate that paliperidone LAI could improve the long-term management of patients with schizophrenia.

